# Cytotoxic Responses Mediated by NK Cells and Cytotoxic T Lymphocytes in Xenotransplantation

**DOI:** 10.3389/ti.2025.13867

**Published:** 2025-02-12

**Authors:** Viktoriia Galdina, Gisella L. Puga Yung, Jörg D. Seebach

**Affiliations:** Department of Medicine, Laboratory of Translational Immunology, Division of Immunology and Allergy, Geneva University Hospitals and Faculty of Medicine, Geneva, Switzerland

**Keywords:** porcine endothelium, NK cells, CD8^+^ T cells, xenograft rejection, cytotoxicity

## Abstract

Xenotransplantation represents a potential solution to the shortage of organs for transplantation. The recent advancements in porcine genetic modification have addressed hyperacute and acute vascular rejection; however, challenges persist with regard to delayed xenograft rejection. Porcine endothelial cells (pECs) represent a crucial target in the context of xenograft rejection, which is mediated by cytotoxic lymphocytes. It is crucial to comprehend the manner in which human natural killer (NK) cells and cytotoxic CD8^+^ T lymphocytes (CTL) recognize and target pECs in order to develop efficacious prophylactic strategies against rejection. The objective of the present review is to synthesize the existing knowledge regarding the mechanisms and techniques employed to modulate xenogeneic responses mediated by human NK cells and CTL. We will elucidate recent methodological advancements, debate potential novel strategies, and emphasize the imperative necessity for further research and innovative approaches to enhance graft survival.

## Introduction

Numerous hurdles in xenotransplantation need to be overcome to achieve tolerance of the recipient’s immune system [1–3]. Hyperacute rejection seems to be resolved due to the availability of knockouts of the major porcine xenoantigens such as αGal [4–6]. Even so, acute vascular and delayed xenograft rejection mediated by both the innate and adaptive immune system remain an obstacle to successful xenotransplantation despite novel systemic immunosuppressive regimens.

The endothelium of a vascularized porcine xenograft represents the initial point of contact with the recipient’s blood, which contains a multitude of immune cell subsets. Consequently, a significant objective of contemporary research initiatives is to evaluate the impact of diverse genetic modifications in porcine endothelial cells (pECs) on human immune cells. It was shown that the complement- and coagulation-mediated damage of pECs can be prevented by knocking out the genes of the three major xenocarbohydrates (αGal, Neu5Gc, and Sda), GGTA1, CMAH, and B4GALNT2, respectively [4], by introducing human complement regulators (hCD46, hDC55, and hCD59) [7, 8], and thrombomodulin (hTBM). Overcoming these hurdles of hyperacute and acute vascular rejection, consistent life-supporting function of xenografted hearts for up to 195 days was achieved in a pig-to-non-human primate (NHP) preclinical model with a combination of αGal knockout (GTKO), and transgenic expression of hCD46 and hTBM [9]. Consistent long-term survival was also demonstrated in pig-to-NHP kidney xenotransplantation using 10GE (genetically engineered) source pigs and FDA-approved immunosuppression [10]. These data provide critical supporting evidence for the safety and feasibility of clinical kidney xenotransplantation, even without CD40/CD154 costimulatory blockade. However, it was not further explored in these models, whether responses mediated by natural killer (NK) cells and cytotoxic CD8^+^ T lymphocytes (CTL) were sufficiently controlled. Further, pig-to-primate experiments are considered as the best-possible pre-clinical constellation for evaluating xenotransplantation, but it is largely unclear to which extent genetic modifications that have been designed for humans remain purposeful in baboons.

The significance of NK cells in xenograft rejection was first shown in the early 90s by Inverardi et al using an *ex vivo* human-to-rat perfusion model [11]. Subsequently, our research group and others have conducted comprehensive, predominantly *in vitro* investigations into the function of NK cells in pig-to-human xenotransplantation [12–14]. A comprehensive review of the immunological barriers, including strategies of inhibition, was published by Sykes and Sachs in 2022 emphasizing approaches to promote and induce T cell tolerance in xenograft recipients [2]. The goal of the present review is to focus on our current understanding of the mechanisms involved in xenogeneic responses mediated by human cytotoxic lymphocytes (NK cells and CTL), in the light of recent advances in the field of xenotransplantation, and to discuss potential strategies for prevention of xenograft rejection.

## Recruitment and Activation of Cytotoxic Lymphocytes by Porcine Endothelial Cells

Antibody binding, complement deposition, and coagulation proteins activate porcine endothelium leading to the release of pro-inflammatory cytokines such as TNF and IL-6. These cytokines subsequently recruit immune cells to the graft [15, 16]. Activated pECs express E- (CD62E) and P- (CD62P) selectin which bind to L-selectin (CD62L) on human lymphocytes, thereby initiating the rolling phase. Human β_2_ integrins, such as LFA-1 (CD11a/CD18), interact with immunoglobulin superfamily members (i.e., porcine ICAM-1) enabling firm adhesion of the lymphocytes [17]. It is of particular significance that the human β1 integrin very late antigen-4 (VLA-4; CD49d/CD29) has the capacity to bind to VCAM-1 (CD106) on pECs [17–19], thereby facilitating NK cell and CTL adhesion. Lastly, it has been demonstrated using transwell assays *in vitro* that transendothelial migration of human leukocytes is dependent on CD99 and the β_2_ integrin CD18 [18]. Conversely, homotypic interactions mediated through CD31 (PECAM-1) have been shown to be incompatible in the pig-to-human context [19].

NK cells and CTL mediate anti-pig cytotoxicity through lytic granule release (perforin and granzyme B) or the Fas/FasL and TRAIL pathways, causing apoptotic and/or necrotic death of the target cells [20–25]. While sharing the killing mechanisms, the robust response of NK cells towards target cells can in turn initiate and amplify CTL-mediated cytotoxicity [26, 27]. Both produce cytokines like interferon-gamma (IFNγ) and tumour necrosis factor (TNF) [28]. In the recent *in vivo* studies of pig-to-NHP and pig-to-human (decedent recipients) solid organ transplantation, NK cells were found to be the early producers of IFNγ [29, 30]. An overview of the classical techniques used to evaluate NK cell and CTL xenoresponses *in vitro* and *ex vivo,* as well as an overview of the current cutting-edge -omics techniques, such as single-cell RNA sequencing (scRNA-seq) and longitudinal RNA-seq analyses, is presented in Box 1. To assess cell-cell interactions, i.e., lymphocytes adhesion to pECs and transendothelial migration under shear stress, our group has recently established a 3D microfluidic system [31], [Tran T et al., in press].

BOX 1 **Methods to analyse NK and CTL porcine xenoresponses.**

***In vitro* (for NK and CTL unless said otherwise)**

**Cytotoxicity release assay.** Here, effector cytotoxic cells are co-cultured with porcine target cells (either pPBMC or pEC) previously loaded with ^51^[Cr] or BADTA which after cellular membrane destruction are released to the culture media and quantified.

**CD107a degranulation assay.** In this flow cytometry-based assay, the degranulation of lytic granule contents by cytotoxic cells upon encounter with porcine target cells is detected by the surface expression of CD107a thanks to the fusion of lytic granule to the cellular membrane. This assay could be coupled to intracellular cytokine detection.

**Adhesion assays using fluorescence time-lapse microscopy.** Porcine endothelial cell destruction is quantified in real-time upon encounter with cytotoxic cells. This method allows in addition to study cytotoxic cell displacement on a carpet of pEC and type of cell elimination. The next level is achieved by using a 3D microvessel system covered by a monolayer of pEC where cytotoxic cells first adhere to encounter and destroy their targets under flow.

**Mix lymphocyte reaction (MLR).** This assay is specific for T cells and measures T-cell responses from the recipient (human or NHP) to xenoantigens. It involves co-culturing responder lymphocytes (recipient) with irradiated or Mitomycin C-treated stimulator cells (donor PBMC) to assess proliferation through thymidine incorporation, CFSE or BrdU staining, or cytokine production by ELISpot or ELISA. Xeno-specific T cells proliferate upon encountering antigens on stimulator cells to which they are specifically reactive, such as SLA-1.

**Transendothelial migration (TEM) assay.** Is used to study the movement of lymphocytes across the endothelial layer. Endothelial cells are cultured in Transwell insert which has a porous bottom that allows immune cells to migrate through it. Lymphocytes migrate into wells containing a chemoattractant or medium and can be quantified once the transmigration is completed.

**Antibody-dependent cell cytotoxicity (ADCC).** This assay measures the activation of NK cells via the CD16 receptor, leading to target cell lysis when IgG xenoantibodies present in the recipient’s sera bind to donor-derived cells. It is performed similarly to the cytotoxicity release assay, where donor cells are labeled beforehand.

**Ex vivo**

 **Biopsies.** Analysis by histology, immunohistochemistry, or immunofluorescence is performed in some cases both during graft acceptance and rejection. This examination evaluates cellular infiltration, tissue architecture, and marker expression to assess immune responses, inflammation, and graft viability. These analyses offer insights into the mechanisms of graft acceptance or rejection, but they are purely descriptive and do not evaluate functional outcomes.

Analysis of CTL and NK cells functions in blood. Here the same assays of cytotoxic release assay, CD107a degranulation, MLR, ADCC.

**Omics era**

 **Bulk and single-cell RNA sequencing.** As a starting material, either biopsies of a xenograft or recipient’s PBMC are used to measure gene expression across the entire cell population (bulk RNAseq) or for each individual cell within the population (scRNAseq). This allows to identify genes of interest which could be targeted in the xenotransplantation setting.

**Spatial transcriptomics of single nucleus/cells.** Combines histology and RNAseq. It enables the mapping of gene expression across different regions of a tissue sample, maintaining spatial context. The method can map the immune response at the site of the xenograft. This includes identifying immune cell infiltration patterns and understanding local cytokine and chemokine expression.

### Natural Killer Cells

**FIGURE 1:**
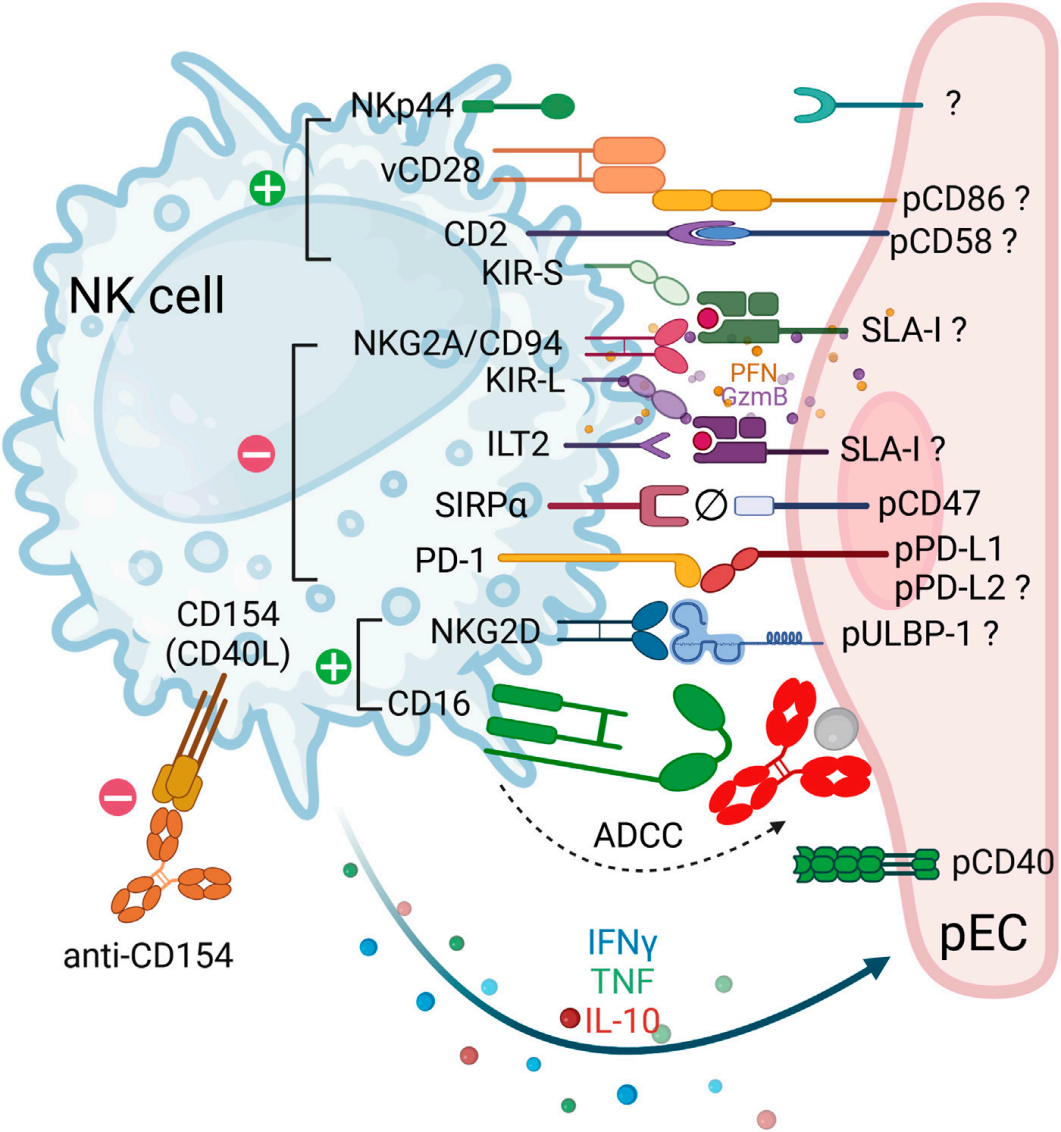
Receptor-ligand interactions between human NK cells and porcine endothelial cells. The cartoon depicts the interactions between human natural killer (NK) cells (blue) and porcine endothelial cells (pEC, pink). In instances where the ligand-receptor pair interaction is functional across species, the corresponding pair is indicated. In cases where the ligand-receptor pair is suspected, or when contradictory data is available, a question mark is added to indicate this. Ligand-receptor interactions that result in either activation or inhibition are represented by green (+) and red (−) circles, respectively. Incompatibility between pCD47 and hSIRPα is symbolized by Ø. The engagement of activating receptors results in the release of lytic granule content (perforins, granzymes and granulysins), which is then followed by the secretion of cytokines (e.g. IFNγ, TNF and IL-10). Co-stimulatory molecules expressed on pEC include CD80/CD86 and PD-L1. NK cells are also capable of expressing these ‘co-stimulatory’ molecules (CD28 and PD-1), thereby regulating their function. Therapeutic strategies that interfere with the costimulatory pathways include anti-CD40L (anti-CD154), as shown in the figure, anti-CD40 and the soluble CTLA-Ig molecule, among others. Finally, NK cells can be strongly activated by the binding of their CD16/FcgRIIIa to antibodies attached to pEC leading to antibody-dependent cellular cytotoxicity (ADCC).

In the context of pig-to-human xenotransplantation, incompatibilities in species-specific molecular interactions can either result in the activation of NK cells or prevent it through a lack of inhibitory signals (Figure 1). NK cells exert their function via two primary mechanisms (i) antibody-dependent cellular cytotoxicity (ADCC) and (ii) direct cytotoxicity. ADCC is initiated when the NK cell receptor CD16 (FcγRIIIA) binds to human antibodies that are directed against xenoantigens expressed on pig endothelium; (ii) in direct cytotoxicity the lack of recognition (“missing self”) of SLA (swine leukocyte antigen) MHC class-I molecules by inhibitory NK cell receptors (CD94/NKG2A, KIRs, and ILT2) [32–34] results in the activation of NK cells. Another immune checkpoint molecule providing inhibitory signals, SIRPα (CD172a), can be expressed on NK cells upon cytokine stimulation [35]. Among different activating NK cell receptors CD2 (LFA-2), NKp44 and NKG2D are involved in both porcine cell lysis and cytokine production [36, 37]. Porcine CD58 may function as an activating ligand for CD2 expressed on human NK cells [38, 39] as evidenced by inhibitory effects of pCD58 blockade on human peripheral blood mononuclear cells (PBMC) cytotoxicity and proliferation in response to porcine cells [40]. The interaction between human NKG2D and porcine ULBP1 (pUBPL1) remains a topic of some debate. The porcine ligand for the activating NK cell receptor NKp44 is still unknown, reported candidates include proliferating cell nuclear antigen (PCNA), heparan sulfate proteoglycans (HSPGs), viral hemagglutinin, MHC class I related chain A (MICA), platelet-derived growth factor (PDGF)-DD, and some HLA-DP molecules [41, 42], however, a widely expressed cellular ligand for NKp44 has remained elusive and controversial. Finally, the impact of glycosylation, in particular sialic acids on the interaction between NK cells and potential target cells needs to be further investigated [43]. The expression of Siglec-7 ligands on target cells can protect them from NK cell-mediated lysis whereas masking of ligands for Siglec-7 and Siglec-9 has been demonstrated to enhance anti-tumour activity of NK cells. This aspect has not yet been evaluated in the context of transplantation.

### Cytotoxic T Lymphocytes

**FIGURE 2:**
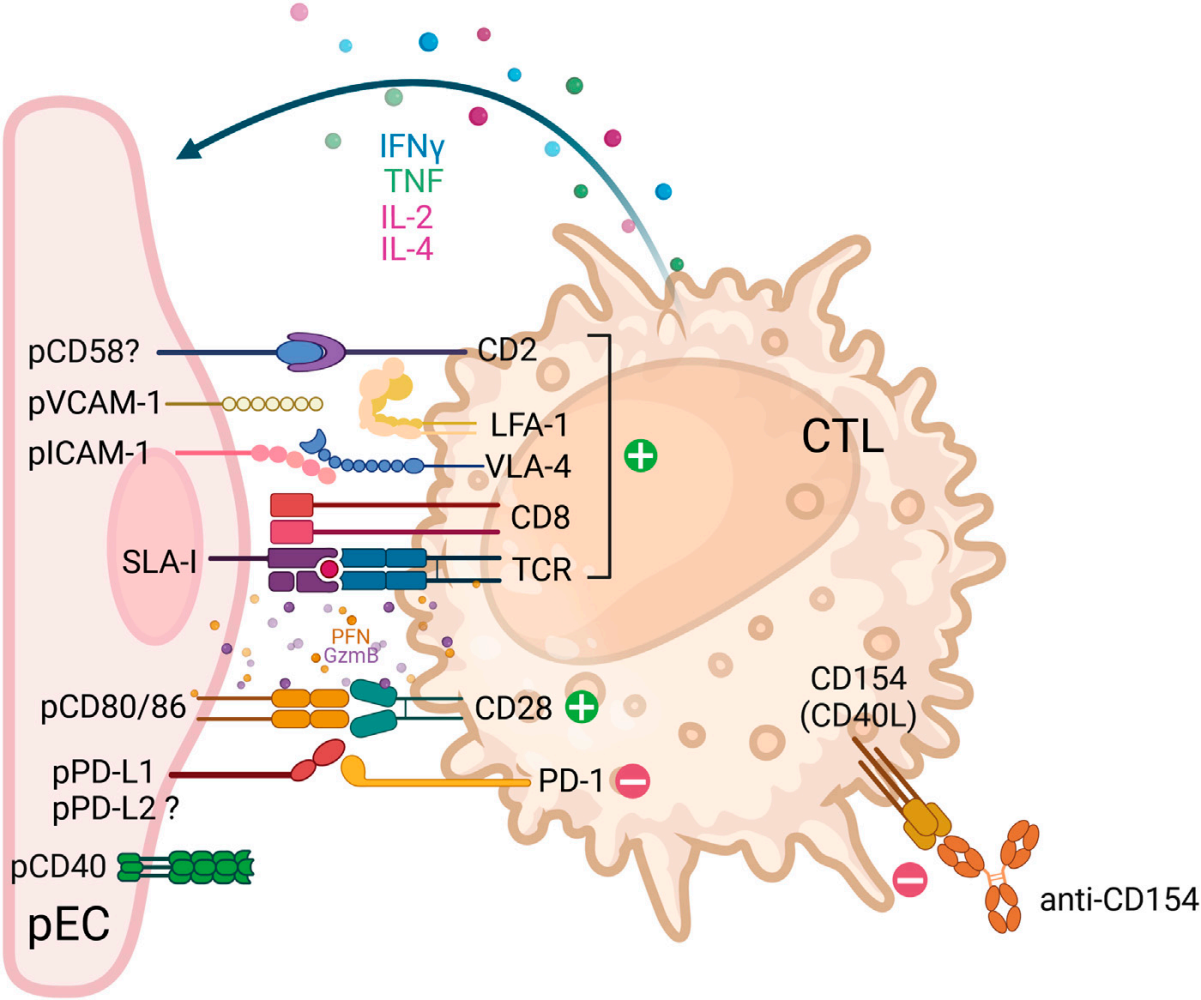
Receptor-ligand interactions between human cytotoxic T lymphocytes (CTL) and porcine endothelial cells. The cartoon depicts the interactions between cytotoxic T lymphocytes (CTL) (CD8+ T cells, orange) with porcine endothelial cells (pEC, pink). In instances where the ligand-receptor pair interaction is functional across species, the corresponding pair is indicated. In cases where the ligand-receptor pair is suspected, or when contradictory data is available, a question mark is added to indicate this. Ligand-receptor interactions that result in either activation or inhibition are represented by green (+) and red (-) circles, respectively. The engagement of activating receptors results in the release of lytic granule content (perforins, granzymes and granulysins), which is then followed by the secretion of cytokines (e.g. IFNγ, TNF, IL-2 and IL-4). The term ‘costimulatory pathways’ was first employed in the context of T cell immunology following TCR engagement (signal 1). The term is used to describe the molecules that are required to further activate or inhibit T cells (signal 2). Such molecules include CD80/CD86 and PD-L1. It is noteworthy that the co-stimulatory CD28 is a dual-function molecule on CTL, capable of binding to CD80/CD86 on pEC, thereby providing activation or to CTLA-4 on helper CD4 T cells, thereby triggering an inhibitory signal. Therapeutic strategies that interfere with the costimulatory pathways include anti-CD40L (anti-CD154), as shown in the figure, anti-CD40 and the soluble CTLA-Ig molecule, among others. Abbreviations: ADCC: antibody-dependent cellular cytotoxicity; CD154: also known as CD40L; CD16/FcgRIIIa: Fc gamma Receptor IIIa; CD2: also known as lymphocyte-function antigen-2 (LFA-2); CTL: cytotoxic T lymphocyte; CTLA-4: cytotoxic T-lymphocyte associated protein 4, also known as CD152 molecule; GzmB: granzyme B; IFNγ: interferon-gamma; IL: interleukin; ILT2: immunoglobulin-like transcript 2, also known as leukocyte immunoglobulin like receptor B1 (LILRB1); KIR: killer cell immunoglobulin-like receptor, -L – inhibitory, -S - activating; LFA-1: lymphocyte function-associated antigen 1, also known as CD11a molecule; NK: natural killer; NKG2A: also known as killer cell lectin like receptor C1 (KLRC1); NKG2D: also known as killer-cell lectin-like receptor K1 (KLRK1); NKp44: natural cytotoxicity triggering receptor 2, also known as NCR2 and CD336 molecule; p: porcine; PD-1: programmed cell death 1 also known as CD279 molecule; PD-L1/2: programmed cell death 1/2 ligand, also known as CD274/CD273; PFN: perforin; SIRPα: signal regulatory protein alpha also known as CD172a molecule; SLA-I: swine leukocyte antigen-1; TCR: T-cell receptor; TNF: tumour necrosis factor; ULBP-1: UL16 binding protein 1; vCD28: variant of CD28; VLA-4: very late antigen-4, integrin formed by alpha4/beta4 subunits (CD49d/CD29 molecules).

Unlike NK cells, naïve CD8^+^ T cells require prior stimulation with porcine antigens target cell elimination [44]. *In vitro* data show that human T lymphocytes recognize porcine cells via both direct and indirect pathways [45–49]. Direct recognition entails T cell receptor (TCR) interactions with SLA-I/II and co-stimulatory molecules CD80/86 [48–52], via CD2 and CD28 pathways [53] on pECs (Figure 2), whereas indirect recognition involves the presentation of pig antigens by recipient’s antigen-presenting cells (APC) [54]. Moreover, *in vitro* studies have demonstrated that the proliferative responses of human T cells are enhanced when both porcine cells and human autologous APC are co-cultured, thereby combining direct and indirect recognition mechanisms [55, 56]. Additionally, indirect presentation was described by the recognition of SLA-I within endothelial cell derived extracellular vesicles [52]. CTL not only recognize SLA-I allelic polymorphisms, but they also require the presence of porcine peptides bound to SLA-I for an efficient xenospecific reaction [57–59]. Consequently, specific T cell clones proliferate and differentiate into effector and then memory cells that react rapidly upon the antigen re-exposure [60]. Co-stimulation can also be provided via pCD86 expressed on porcine cells binding CD28 on both CTL and NK cells [61–64], as well as binding of CD154 (CD40L) and CD40 [65, 66] (Figures 1, 2). It is noteworthy that *in vitro* studies utilizing human and baboon PBMC demonstrated a reduction in T cell responses to GGAT1 KO pig-derived pEC in comparison to wild-type pEC, irrespective of whether the pEC were activated. CD4 and CD8 lymphocytes showed reduced proliferation, cytokine production, and granzyme/perforin content. Nevertheless, the specific mechanisms by which αGal modulates the T cell response remain to be elucidated [67].

### Findings in the Recent Studies *In Vivo*

The findings across various animal models demonstrated the complexity and challenges associated with NK and CTL responses in pig-to-primate xenotransplantation. In baboons that received porcine heart transplants expressing either hCD55 or hCD46, without specific T cell depletion, the rejection process occurred rapidly, within 5–7 days, and was characterized by a high prevalence of macrophages and T cells [68, 69]. Similarly, porcine islet transplantation into cynomolgus monkeys and rhesus macaques resulted in infiltration and rejection, which were primarily mediated by T cells, even before macrophage recruitment into the tissues [70, 71]. In the case of porcine cartilage transplanted into cynomolgus monkeys, up to 90% of the infiltrating cells were T cells, with equal amounts of CD4^+^ and CD8^+^ T cells, as observed 1 month post-transplantation [72]. Recent studies have demonstrated the involvement of NK cells and a few adaptive CD3^+^ cells in porcine kidney xenografts transplanted into brain-dead recipients. NK cells and T cells were identified in the glomeruli 54 h post-transplantation, with transcriptome profiling showing increased expression of genes related to IFNγ response, NK cells (*HLA-E* and *FCGR3A/B*), and T cell development (*CD81*) [73]. Longitudinal bulk RNA-seq of kidney xenografts revealed increased IFNγ signaling as early as 12 h post-transplantation, predominantly attributed to infiltrated NK cells [30]. Single-cell RNA-sequencing of recipient’s PBMCs revealed two distinct waves of immune response with upregulation of genes associated with NK and T cells. This correlated with increased IFNγ expression in NK cells at 12 and 48–53 h [30]. Similar trends were observed through scRNA-seq analysis on day 38 in the first pig-to-human cardiac xenograft recipient. Dominant changes in immune cell type composition across the time points were observed for monocytes, both CD4^+^ and CD8^+^ T cells, and NK cells, accompanied by the upregulation of genes related to NK and T cell activation, and IFN responses. However, it was difficult to pinpoint any single etiology for the xenograft dysfunction [74]. The latest multi-omics analyses of samples from two deceased human recipients of 10-gene-edited pig hearts have revealed two distinct scenarios regarding the administration of thymoglobulin and the duration of cold ischemic time. One of the decedents exhibited a lack of evidence of immune cell activation, whereas the other decedent displayed an increase in T and NK cells in both circulating PBMC and xenograft [75]. This notable rise in lymphocytes in one of the recipients may be attributed to the longer ischemic time of the pig heart and the T cell depletion protocol resulting in the expansion of memory T cells and rapid xenograft injury.

## Strategies of Inhibition

### NK Cells

Based on the regulation of NK cell function predicted by the “missing self” theory and the observation that SLA MHC class I molecules do not appear to interact sufficiently with inhibitory human NK cell receptors, human MHC class I molecules were introduced into pig cells to protect against human NK cytotoxicity [12, 13, 76–78] (Table 1). For example, HLA-E is a low polymorphic, non-classical HLA class I molecule and a major ligand for the inhibitory receptor CD94/NKG2A, which is expressed on a subset of NK cells. Thus, expression of HLA-E on pig endothelial cell at least partially prevents direct human NK cytotoxicity. This finding was confirmed in *in vitro* studies [79, 80] and in *ex vivo* perfusion studies of porcine limbs, hearts and lungs with human blood [8, 76, 81]. In addition, decreased proliferation of both human T and NK cells was demonstrated *in vitro* in response to fibroblasts derived from GTKO pigs expressing HLA-G1^+^. Furthermore, transplantation of islets from GTKO/HLA-G1^+^ pigs into diabetic mice resulted in restored normoglycemia [77]. Several other studies have confirmed the partial inhibition of NK cell cytotoxicity, but also of adhesion, by HLA-G1 expressed on pEC *in vitro* [96–98], including soluble HLA-G1 [99]. Finally, higher levels of protection have been achieved by co-expressing HLA-E and HLA-G1 *in vitro* assays, primarily by stabilizing HLA-E expression [82, 83]. However, complete protection from xenogeneic NK cytotoxicity has not yet been achieved, probably due to the heterogeneity of the inhibitory NK cell receptor repertoire.

**TABLE 1:** Strategies of inhibition of human cytotoxic lymphocytes tested to date.

Genetic modification	Model	Inhibited lymphocytes	Reference
HLA-E	*Ex-vivo* perfusion of pig hearts	NK cells	[76]
	*In vitro*		[79, 80]
	*Ex-vivo* perfusion of pig limbs		[8]
	*Ex-vivo* perfusion of pig lungs		[81]
Co-expressing HLA-E and HLA-G1	*In vitro*		[82, 83]
pULBP1 KO	*In vitro*		[84,85]
Beta-2 microglobulin (KO B2M)/SLA-I low	*In vitro*	CD8^+^ T cells	[86]
Membrane-bound human FasL and human decoy Fas	*In vitro*; pig-to-rat islets Tx		[21, 87]
Porcine cellular FLICE inhibitory protein (c-FLIP)	*In vitro*; pig-to-rat TG PAEC Tx under kidney capsules		[88]
HLA-G1	*In vitro*; pig-to-mouse islets Tx	T cells, NK cells	[77]
hCD152 (hCTLA4-Ig)	*In vitro*; pig-to-mouse TG PAEC Tx under kidney capsules	T cells, NK cells	[64, 89]
	*In vitro*; pig-to-rat skin Tx	PBMC	[90]
LEA29Y^a^	*In vitro;* pig-to-mouse islet Tx	T cells	[91,92]
hPD-L1	*In vitro*	T cells, NK cells	[93, 94]
	*In vitro; Ex-vivo* kidney perfusion	T cells (CD4^+^ and CD8^+^)	[95]

^a^ LEA29Y is an affinity-optimized derivative of hCTLA4-Ig.

The role of *α*Gal in recognition of porcine target cells by human NK cells was extensively reviewed in 2017 [12]. In short, human NK cytotoxicity against αGalKO pEC in the absence of Abs was equivalent to that in αGal expressing cells. On the other hand, interactions between *α*Gal and the activating NK cell receptor CD161 remains a matter of debate [100–102]. The deletion of *α*Gal might result in the recognition of terminal epitopes and *N*-acetyllactosamine (NAcLac) [100] by NK cells expressing CD161, however, the significance of such neoantigens needs further investigation. Immortalized pEC with gene KO for the enzymes of the three major xenoAg sugars, GGTA1, CMAH and B4GALNT2, with or without deletion of SLA-I α-chain/β-2, strongly induced degranulation of IL-2-activated human NK cells [103]. The results indicate that other so far unidentified activating ligands of carbohydrate and/or protein nature, are involved in human anti-pig NK cytotoxicity. The question of the exact ligands on porcine cells stays unsolved. However, some of these unidentified ligands can be masked by dextran, an analog of heparan sulfate proteoglycan, which is a key molecule for the anticoagulant and anti-inflammatory properties of the resting (non-activated) endothelium, providing partial protection against human NK cytotoxicity [104].

Human NK cell receptors NKp44 and NKG2D are activated by ligands present in pECs [37] and porcine chondrocytes [105]. Although porcine ULBP1 has been confirmed as a ligand for human NKG2D, its removal did not result in complete inhibition of NK cell cytotoxicity, indicating the potential involvement of additional ligands [106]. Using CRISPR technology, more recent findings demonstrated that pULBP1 gene knockout in porcine endothelial cell lines or transgenic pigs was not sufficient to fully suppress NK cell degranulation and cytotoxicity [84, 85].

Some other alternative and indirect methods of NK cell inhibition were explored *in vitro*. Controversial data have been reported on the protective effect of FasL expression on porcine endothelial cells [21, 87, 107]. An effective strategy to mitigate macrophage responses is the transgenic expression of CD47 on porcine cells, which acts as a “don’t eat me” signal via the receptor SIRPα and inhibits phagocytosis [108–110]. Of note, CD47 was present in the 10GE pig hearts used in the first pig-to-human xenotransplantations. It appears that it did not entirely prevent macrophage responses, as CD68 staining demonstrated clusters of macrophages on POD34. However, CD47 gene expression in the heart decreased over time, as other transgenes (HO1, CD46, and DAF) also showed lower expression, raising the question of how effective and robust the genetic modifications were [74]. Since NK cells can also express SIRPα they could potentially be inhibited via this pathway. Indeed, expression of CD47 on HLA-deficient target cells inhibited SIRPα-expressing NK cells *in vivo* in a mouse model and *in vitro* using a CD47 transduced NK target cell line (K562) [35]. These recent results need to be confirmed in the pig-to-human and pig-to-NHP settings.

### Cytotoxic T Lymphocytes

As mentioned above, T cells recognize porcine cells directly restricted by MHC molecules; therefore, elimination of SLA class I expression on pig cells should protect from CD8^+^ T lymphocyte-mediated lysis. Indeed, cells obtained from engineered pigs knocked down for beta-2 microglobulin (KO B2M) and expressing an SLA-I^low^ phenotype were less capable in triggering proliferation of human PBMC *in vitro*, which was mainly due to the non-responsiveness of CD8^+^ T cells. However, no difference in cytotoxicity exerted by human PBMC or purified NK cells was observed [86]. Alternatively, as shown in a recent *in vitro* study, silenced expression of SLA-I and -II on porcine kidney epithelial cells resulted in weak CTL reactivity and cytokine secretion but, surprisingly, did not trigger NK cell degranulation [111]. Therefore, further investigation is required to gain a deeper comprehension of the impact of SLA knockouts or silencing on CTL and NK cell responses. This is crucial to prevent the potential exacerbation of the missing-self problem.

One of the ways to prevent delayed xenograft rejection (DXR) orchestrated by adaptive immunity is the administration of immunosuppressive therapy (see below), including blocking co-stimulatory molecules of T cell – APC interactions (i.e., CD80/CD86-CD28) with CTLA4-Ig (Abatacept) or its affinity-optimized derivative LEA29Y (Belatacept) [112]. To circumvent the drawbacks of systemic immunosuppression, these inhibitory molecules have been expressed transgenically in pig cells with the objective of enabling local protection of the xenograft from CTL [91]. It was shown that serum from LEA29Y [92] and hCTLA4-Ig [90] transgenic pigs reduced the proliferation of T cells in a mixed-lymphocyte reaction compared to wild-type (WT) controls. Another similar approach to protect xenograft is by expression of human programmed death-ligand 1 (hPD-L1) on porcine cells, which diminished human T cell- and NK cell-mediated cytotoxicity and proliferation *in vitro* [93, 94]. In a recent study, human T cells were perfused through porcine kidneys expressing hPD-L1 *ex vivo*. T cells adhered similarly to the endothelium and infiltrated the tissue of both WT and transgenic kidneys, but secretion of IFNγ, TNF, granzyme B, and perforin was decreased. The inhibition of T cell activity was confirmed *in vitro* by co-culturing renal pECs in proliferation and cytotoxicity assays [95]. The expression of inhibitory molecules proved to be quite efficient in both *in vitro* and *ex vivo* experiments. However, the confirmation of these findings *in vivo* remains to be seen.

The Fas/FasL pathway utilized by cytotoxic cells presents another potential target for inhibiting NK and CTL responses. Overexpressing membrane-bound human FasL and a human decoy Fas in pECs led to partial protection from CTL-mediated cytotoxicity [21]. However, protection from NK cell cytotoxicity was not achieved *in vitro*, in contrast NK cell migration through pECs and chemotaxis of human polymorphonuclear cells were strongly increased by the expression and cleavage of soluble FasL [107]. As an inhibitor of Fas-receptor signaling, overexpressed porcine cellular FLICE inhibitory protein (c-FLIP) in pECs resulted in a significant reduction of CTL-mediated lysis, with up to 75% suppression confirmed *in vitro* and prolonged graft survival in a rat xenotransplantation model [88].

The question of tolerance induction arises. It would be advantageous to induce tolerance of the innate and adaptive immune systems in order to prevent rejection while maintaining the ability of the immune system to protect the transplant recipient and the graft from infection. One approach to induce recipient tolerance and to avoid heavy immunosuppression could involve porcine thymus transplantation. Studies in a pig-to-humanized mouse model have demonstrated its efficacy in promoting tolerance both in mixed leukocyte reactions [113] and xenograft settings [114]. However, efficient positive selection of human T cells in the porcine thymus remains a challenge [115]. Porcine dendritic cells are likely to be pivotal cells in the initiation of CTL responses [116]. An alternative approach to achieving T-cell tolerance is therefore to use tolerogenic or regulatory dendritic cells (DC), or porcine mesenchymal stromal cells (pMSC) aiming to prevent indirect recognition [54, 117–120].

### Co-Stimulation Blockade and Immunosuppressive Regimens

Costimulation blockade to inhibit xenogeneic NK and CTL responses can be achieved by two major approaches: (i) blocking receptor-ligand interactions between human or porcine APC and human T cells, using pharmacological compounds such as Abatacept/Belatacept or antibodies directed against CD40 or CD154, or (ii) by transgenic expression of genes with an inhibitory effect on costimulation in pig endothelial cells (surface expression and secretion) such as hCD152 (CTLA4 or PD-L1). Blocking of pCD86, a ligand of the activating costimulatory receptor CD28, combined with αGal downregulation, resulted in complete protection from human NK cell cytotoxicity [64]. In a previous study, Costa and colleagues demonstrated that the expression of the inhibitory costimulation receptor hCD152 (hCTLA4-Ig) on pECs resulted in the inhibition of T cell and NK cell activation [64]. The same group later developed a pig-to-mouse xenotransplantation model where they implanted pECs transduced with hCD152-hCD59 under the recipients’ kidney capsules. Enhanced survival for up to 5 weeks was observed in modified xenografts compared to the control group [89]. Simultaneous blocking of NK cell activating receptors CD2 and NKG2D resulted in the suppression of NK-mediated killing *in vitro* [36]. The blockade of the CD40^−^CD154 co-stimulation pathway using therapeutic monoclonal antibodies was reported to be successful in pig-to-NHP kidney transplantation [121], and in pig-to-NHP corneal transplantation, the xenografts exhibited prolonged survival (more than 933 days) [121]. This outcome was particularly attributable to the reduction in cellular infiltrates, including CTL and macrophages, as well as the diminished levels of cytokines (i.e., IFNγ, TNF, IL-2) [122]. Given that activated NK cells express CD154 (CD40L), and that binding to CD40 provides a positive signal leading to cytotoxicity [65, 123], the blockade of CD154 signaling may also be beneficial in attenuating NK cell responses. However, this hypothesis has yet to be investigated. Concurrently, the simultaneous blockade of CD28 and CD40^−^CD154 pathways resulted in the complete abrogation of T cell proliferation when co-cultured with porcine APCs in a preliminary report [124]. Conversely, the cross-species interaction of CD154-pCD40 resulted in pEC activation, with the induction of E-selectin (CD62E), VCAM-1 (CD106) and SLA class-II [125].

The use of an immunosuppressive (IS) regimen is essential for prolonging xenograft survival in pig-to-NHP models despite the utilization of genetically modified porcine tissues and organs. For example, IS consisting of cyclosporin A, cyclophosphamide, and methylprednisolone delayed rejection in baboons and rhesus monkeys [68, 126]. Other experiments using hCD46 transgenic pig hearts and liver hDAF (hCD55) xenografts resulted in rejection characterized by infiltrates with CD4^+^ and CD8^+^ T cells [69]. Since those experiments were performed without prior T cell depletion, the effects of CTL were detrimental quite early after transplantation. T cell depletion showed benefits for renal graft survival (>400 days) in rhesus macaques [127]. Interestingly, these results were achieved by selected targeting of CD4^+^ but not CD8^+^ T cells, which could be explained by the failure of CD8^+^ T cells to exert their function in the absence of helper T cells. Although this approach initially proves effective, CD8^+^ T cells proliferate rapidly following T cell depletion therapies, with the majority comprising effector-memory T cells [128]. This was recently confirmed in a pig-to-baboon genetically modified porcine kidney and heart transplantation models [10, 129–131]. In compassionate pig-to-human cardiac xenotransplantation, IS regimen based on pig-to-baboon studies (induction with ATG and anti-CD20, anti-CD40, MMF, tacrolimus) was insufficient to provide long-term survival, presumably due to patients’ comorbidities [74]. Rapamycin, an mTOR-inhibitor exhibits several properties that may confer particular benefit in the context of xenotransplantation, including inhibition of T cell proliferation, promotion of the number of T regulatory cells, prevention of pig graft growth, and anti-inflammatory, anti-viral and anti-cancer effects [132, 133]. Whereas the use of rapamycin, a mTOR pathway blocker used as a maintenance IS treatment, has been observed *in vitro* to interfere with the xeno-specific T cell proliferative responses to pEC, in addition to modulating the pEC immunogenicity via a PD-1/PD-L1 independent pathway. Under the same conditions, belatacept only partially inhibited xenogeneic T cell proliferation and reduction of CD2 expression [134].

With regard to the impact of IS regimens on NK cells, our findings indicate that a range of NK cell functions can be suppressed by IS drugs (such as methylprednisolone and therapeutic immunoglobulins) *in vitro* [135]. Recently, clinical trials of a therapeutic CD38 antibody for antibody-mediated rejection in kidney allotransplantation have demonstrated a transient depletion of CD16^bright^ NK cells and improved graft function. These results may potentially be implemented in the context of xenotransplantation [136].

## Concluding Remarks and Future Perspectives

In recent years, xenotransplantation has made significant progress, especially with the advent of genetic modifications designed to attenuate hyperacute rejection. Notwithstanding these advances, acute vascular rejection and delayed xenograft rejection continue to present substantial challenges, driven by intricate interactions between the recipient’s innate and adaptive immune systems and the xenograft. Genetic modifications have shown efficiency in mitigating several immune responses, yet they fall short in providing comprehensive protection from NK and CTL-mediated cytotoxicity.

In conclusion, there are multiple pivotal areas within the field of xenotransplantation research that warrant further investigation. First, additional combinations of genetic modifications to porcine tissues should be explored, such as more effective expression of human non-classical MHC class I molecules (HLA-E, HLA-G1) in addition to targeting SLA class I expression, with the aim of better suppressing NK and CTL responses. Of particular interest would also be combinations of other inhibitory pathway transgenes, such as co-expression of HLA-E, CD47, PD-L1, and so forth. Secondly, the utilization of multi-omics analyses will likely offer deeper insights into the immune response at various stages following xenotransplantation, which may ultimately result in the identification of novel therapeutic targets. Thirdly, research into local immunosuppression techniques, such as the expression of human PD-L1 or LEA29Y on xenografts, could help in minimizing systemic immunosuppression and enhancing the long-term viability of xenografts. Future work will likely also explore novel combinations of co-stimulation blockade with organs from transgenic pigs to manage T cell and NK cell activation more effectively. Allotransplantation trials will provide new insights generating strategies for addressing antibody- and cellular-mediated rejection. Another avenue for exploration is the role of glycosylation in cellular responses and the potential of inducing recipient tolerance, for example, through thymus transplantation, thereby reducing the need for lifelong immunosuppression. Ultimately, understanding and addressing the complexities of both innate and adaptive immune responses will be crucial to overcoming the remaining barriers in xenotransplantation and advancing its successful clinical application.
